# PHOX2B-Mediated Regulation of *ALK* Expression: *In Vitro* Identification of a Functional Relationship between Two Genes Involved in Neuroblastoma

**DOI:** 10.1371/journal.pone.0013108

**Published:** 2010-10-01

**Authors:** Tiziana Bachetti, Daniela Di Paolo, Simona Di Lascio, Valentina Mirisola, Chiara Brignole, Marta Bellotti, Irene Caffa, Chiara Ferraris, Michele Fiore, Diego Fornasari, Roberto Chiarle, Silvia Borghini, Ulrich Pfeffer, Mirco Ponzoni, Isabella Ceccherini, Patrizia Perri

**Affiliations:** 1 Laboratory of Molecular Genetics, G. Gaslini Children's Hospital, Genoa, Italy; 2 Experimental Therapy Unit, Laboratory of Oncology, G. Gaslini Children's Hospital, Genoa, Italy; 3 Department of Pharmacology, School of Medicine, Università degli Studi di Milano and CNR-Institute of Neuroscience, Milan, Italy; 4 Advanced Molecular Diagnostics, National Cancer Research Institute, Genoa, Italy; 5 Dept. Biomedical Sciences and Human Oncology, University of Turin, Turin, Italy; Roswell Park Cancer Institute, United States of America

## Abstract

**Background:**

Neuroblastoma (NB) is a severe pediatric tumor originating from neural crest derivatives and accounting for 15% of childhood cancer mortality. The heterogeneous and complex genetic etiology has been confirmed with the identification of mutations in two genes, encoding for the receptor tyrosine kinase Anaplastic Lymphoma Kinase (*ALK*) and the transcription factor Paired-like Homeobox 2B (*PHOX2B*), in a limited proportion of NB patients. Interestingly, these two genes are overexpressed in the great majority of primary NB samples and cell lines. These observations led us to test the hypothesis of a regulatory or functional relationship between *ALK* and *PHOX2B* underlying NB pathogenesis.

**Methodology/Principal Findings:**

Following this possibility, we first confirmed a striking correlation between the transcription levels of *ALK*, *PHOX2B* and its direct target *PHOX2A* in a panel of NB cell lines. Then, we manipulated their expression in NB cell lines by siRNA-mediated knock-down and forced over-expression of each gene under analysis. Surprisingly, *PHOX2B*- and *PHOX2A*-directed siRNAs efficiently downregulated each other as well as *ALK* gene and, consistently, the enhanced expression of *PHOX2B* in NB cells yielded an increment of ALK protein. We finally demonstrated that *PHOX2B* drives *ALK* gene transcription by directly binding its promoter, which therefore represents a novel *PHOX2B* target.

**Conclusions/Significance:**

These findings provide a compelling explanation of the concurrent involvement of these two genes in NB pathogenesis and are going to foster a better understanding of molecular interactions at the base of the disease. Moreover, this work opens new perspectives for NBs refractory to conventional therapies that may benefit from the design of novel therapeutic RNAi-based approaches for multiple gene targets.

## Introduction

Novel insights into the molecular pathogenesis of neuroblastoma (NB), a severe pediatric tumor originating from neural crest cells and accounting for 15% of childhood cancer mortality, have been gained after the identification of germline as well as somatically acquired mutations in the genes encoding the paired-like homeobox 2b (*PHOX2B*) transcription factor [1], [2] and the Anaplastic Lymphoma Kinase (*ALK*) tyrosine kinase receptor [3].

The *PHOX2B* gene is involved in the specification of the noradrenergic phenotype during the development and differentiation of neural crest derivatives [4]–[9]. Missense and frameshift mutations of this gene were identified in only a few pedigrees of familial NB [1], [2], [10], [11] and in about 4% of sporadic cases [12], suggesting genetic heterogeneity of NB [13]. *PHOX2B* mutations are often found in association with other neurocristopathies such as Congenital Central Hypoventilation Syndrome (CCHS) and Hirschsprung disease (HSCR), likely modifying susceptibility to NB in the corresponding patients [1], [2], [10], [11], [13]. Moreover, the involvement of *PHOX2B* and its paralogue *PHOX2A* in NB pathogenesis seems to be also mediated by a mechanism of gene up-regulation [14], with abundance of *PHOX2B* transcript shown to be highly prognostic of poorer progression-free and overall survival [15], [16]. Little is known about physiological regulation of the *PHOX2* genes transcription, except that *PHOX2B* expression depends on an auto-regulatory mechanisms in NB cells [17] and regulates transcription of *PHOX2A* [6]. Other known transcriptional targets of PHOX2B are *TH* (Tyrosine Hydroxylase) and *DBH* (Dopamine-Beta-Hydroxylase), two genes encoding enzymes involved in the cathecolamine biosynthesis [5], [18], TLX-2, a transcription factor controlling development of enteric innervation [9], *RET*, the major gene involved in the complex inheritance of HSCR [19] and *MSX-1*, a negatively regulated homeobox gene [20].

More recently, mutations associated with both hereditary and sporadic neuroblastoma were discovered in the *ALK* tyrosine kinase [3], [21], a gene mapping to a region previously found in linkage with NB [3], [22]. The *ALK* gene was already known to have a physiological role in neuronal development [23] and to be involved in the pathogenesis of cancer, especially lymphomas but also solid tumors of ectodermal, myofibroblastic or neuroblastic origin [23]–[25]. About 11–12% of the NB tumors were shown to carry non-synonymous sequence variations in conserved positions of the tyrosine kinase domain. Particularly, the most frequent mutant ALK proteins p.F1174L and p.R1275Q demonstrated gain-of-function kinase activity [3], [21], [26]–[27]. Molecular events able to promote *ALK* gene transcription may also have a pathogenetic role but only 3–4% of NB cases were found to bear extensive *ALK* amplification while 17–23% presented lower levels of *ALK* gene gain (2≤gene copies ≤4) [3], [21], [26]–[30], therefore most of the ALK over-expression in NB still remains unexplained.

Functional assays showed induction of a constitutive kinase activity in overexpressed and/or hyperphosphorylated ALK proteins, either mutated or wild type. Accordingly, the knock-down of *ALK* expression in cell model systems led to a marked decrease of cell proliferation clearly indicating ALK as a critical player in NB development [3], [21], [26]–[27]. Notably, *ALK* mutations and amplifications as well as gene over-expression were found to significantly correlate with other unfavorable features of poor outcome in advanced/metastatic compared with localized tumors [3], [21], [26]–[29]. Details on regulatory molecular mechanisms acting under physiological conditions or sustaining over-expression of the *ALK* gene in NB are currently unknown.

Therefore, i) *PHOX2B* and *ALK* mutations are involved either in the initiation or the progression of NB and ii) wild-type as well as mutated transcripts of both these genes are reported to be overexpressed in the vast majority of the NB cell lines and tumor samples analyzed. This suggests their possible concurrent role in the development and/or maintenance of the sympathetic nervous system, thus prompting us to test the hypothesis of a cross-talk between *PHOX2B* and *ALK*.

Here, a number of compelling evidences are reported, demonstrating extensive co-regulated over-expression of *PHOX2A*, *PHOX2B* and *ALK* in NB cell lines and a novel PHOX2B-mediated effect on *ALK* transcriptional induction, sustained by *PHOX2B* binding the *ALK* promoter region, thus establishing *ALK* as a novel PHOX2B target gene.

## Results

### Correlated expression of *ALK*, *PHOX2B* and *PHOX2A* in NB cells

To investigate correlations between *ALK* and *PHOX2B* expression, we first carried out transcription analysis by Real-time RT-qPCR in a panel of 13 NB cell lines and additional control samples (see below). Based on the reported expression levels of *PHOX2B* and *PHOX2A* in NB cells [14], we also analyzed the latter gene.

**Figure 1:**
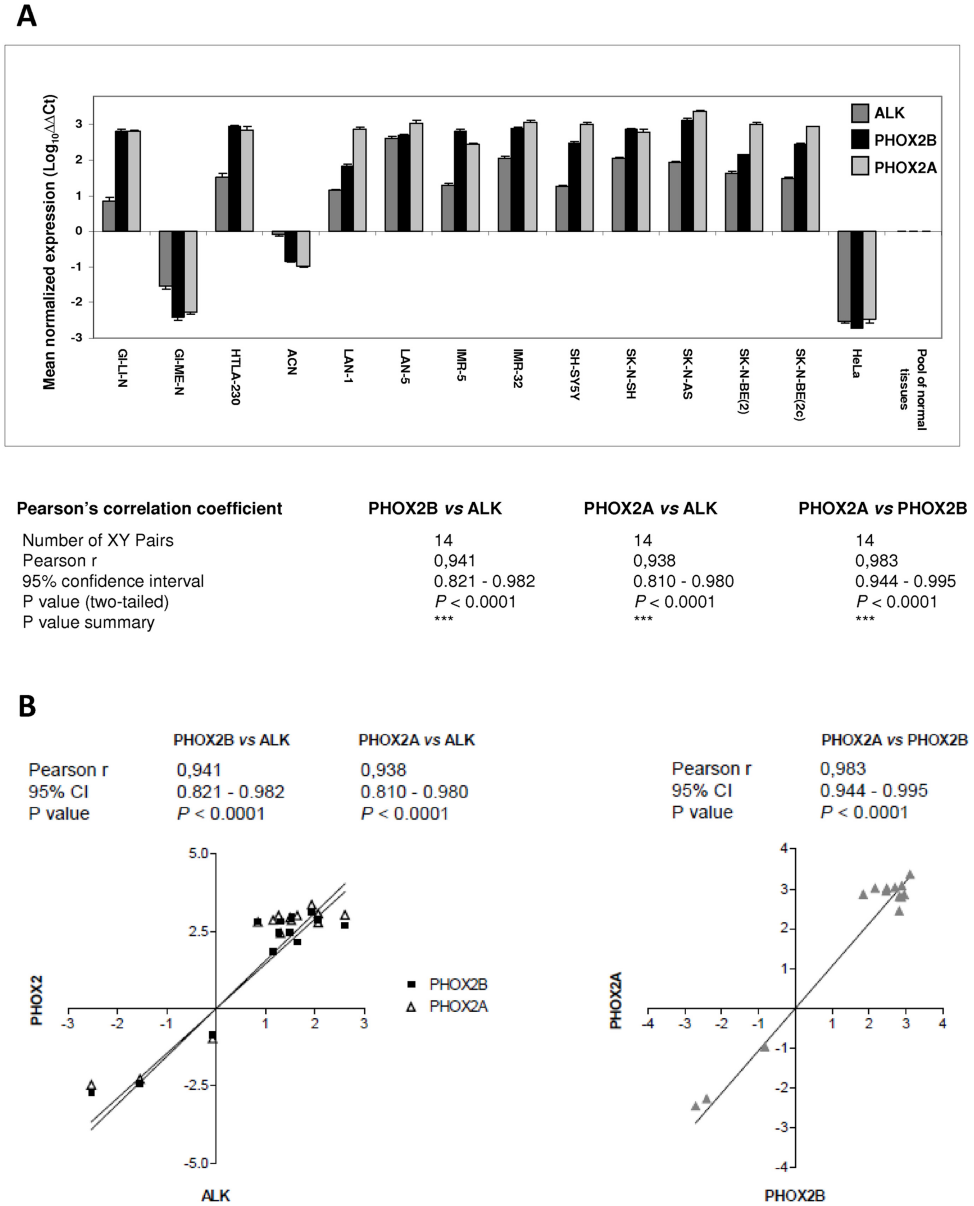
Gene expression analysis in NB and HeLa cells and correlation analysis. (A) Relative gene expression analysis of the *ALK*, *PHOX2B* and *PHOX2A* genes, carried out in a panel of NB and in HeLa cell lines by real-time RT-qPCR using a pool of normal tissue RNAs as reference sample (see Methods), shows over-expression of the three genes in all but two NB cell lines tested (GI-ME-N and ACN). (B) X-Y Plots showing a significant correlation between the expression level of *PHOX2B* and *PHOX2A vs.ALK* (left) and *PHOX2Avs. PHOX2B* (right) genes in the analyzed cell lines. Pearson's correlation coefficient indicates a very significant correlation of the three transcription levels *vs.* each other. Values are the mean ± s.d. of N = 3 independent RT-qPCR analyses performed in triplicate.

*ALK*, *PHOX2B* and *PHOX2A* resulted to be highly expressed in almost all analyzed NB cell lines (Figure 1) with respect to a pool of normal tissues and to HeLa cells, a cervix carcinoma cell line characterized by low level of *ALK* expression [31] and almost undetectable expression levels of the two *PHOX2* genes [6]. Pearson's correlation coefficients (r) confirmed strongly related transcriptional levels among the three genes [*PHOX2B vs. ALK* (r = 0.941; *P*<0.0001), *PHOX2A vs. ALK* (r = 0.938; *P*<0.0001) and *PHOX2A vs. PHOX2B* (r = 0.983; *P*<0.0001)] in all samples tested (Figure 1).

To investigate on the possible inter-regulated transcription of these three genes, we considered to manipulate *ALK, PHOX2B* and *PHOX2A* expression *in vitro*, performing experiments of either knock-down or forced-expression for each of them.

### *ALK* expression is regulated by *PHOX2* genes

**Figure 2:**
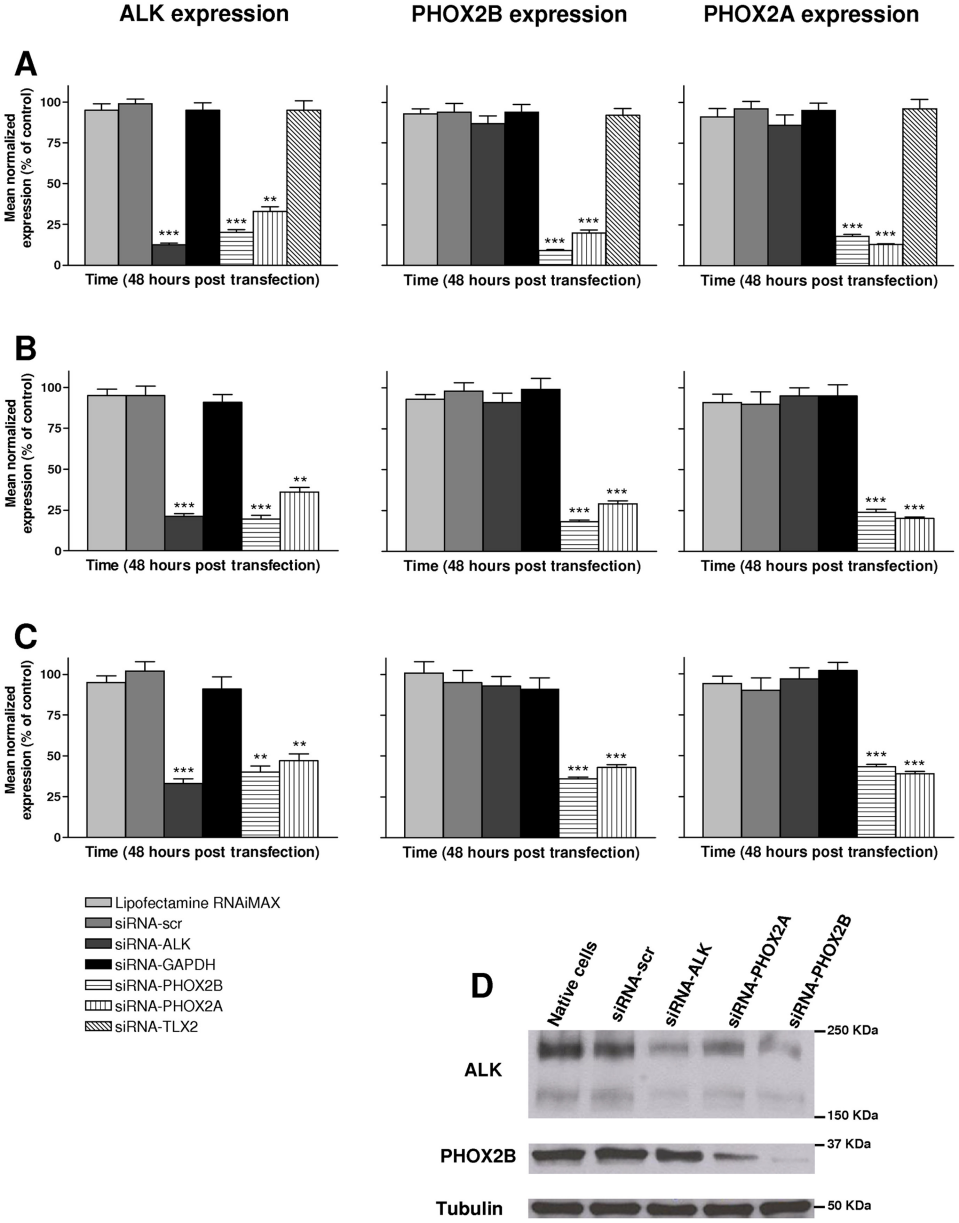
siRNA-mediated silencing of *ALK, PHOX2B* and *PHOX2A* in NB cells. Effects on the transcription level of the *ALK* (left side graphs), *PHOX2B* (middle graphs) and *PHOX2A* (right side graphs) genes after knock-down of the same genes in SHSY-5Y (A), IMR-32 (B) and HTLA-230 (C) cells. Gene-specific knock-down, evaluated 48 hours post-transfection by real-time RT-qPCR analysis, is very effective but also *PHOX2*-directed siRNAs are able to downregulate *ALK* at a similar extent (**: *P*<0.01; ***: *P*<0.001). Values are the mean ± s.d. of N = 3 independent experiments performed in duplicate. (D) Gene silencing was confirmed at 72 hours post-transfection by Western blot.

*ALK, PHOX2B* and *PHOX2A* silencing was achieved through gene-directed siRNA in three NB cell lines, namely IMR-32, HTLA-230 and SH-SY5Y, this latter carrying a p.F1174L ALK mutation, showing a high expression of the three genes. The efficiency of gene silencing as well as the downstream effects of RNA interference on other genes was evaluated by Real-time RT-qPCR at 24, 48 and 72 hours post-transfection. The most powerful sequence among three siRNAs tested for each targeted gene, was used. A scrambled sequence and a siRNA directed against the *GAPDH* gene were used as controls in three distinct experiments, each performed in duplicate. Gene specific silencing was very effective already at 48 hours post-transfection, ranging between 87% and 91% for the three genes under analysis (*P*<0.001 when compared to scrambled siRNA) in SH-SY5Y (Figure 2A). In particular, *PHOX2B*-directed siRNA downregulated *PHOX2A* expression (82%, *P*<0.001) and *PHOX2A*-siRNA lowered *PHOX2B* (80%, *P*<0.001) expression, suggesting reciprocal regulation of these two paralogous genes. Interestingly, *ALK* expression resulted decreased following *PHOX2B* (80%, *P*<0.001) and *PHOX2A* (67%, *P*<0.01) silencing. In contrast, *ALK*-siRNA did not alter *PHOX2A* or *PHOX2B* mRNA levels, thus showing a unidirectional mechanism of transcriptional regulation where *PHOX2* genes control *ALK* expression. Finally, as expected, the silencing of *TLX2*, a gene known as a downstream target of the *PHOX2* genes [9], did not affect the expression level of the studied genes. Similar results were obtained by transfection in IMR-32 (Figure 2B) and HTLA-230 cell lines (Figure 2C). Western blot analysis carried out on total proteins extracted 72 hours post-transfection confirmed a PHOX2-mediated down-regulation of ALK (Figure 2D, lane 5). Moreover, in agreement with the mRNA levels, *ALK* knock-down did not result in altered expression of PHOX2B protein (Figure 2D, lane 3).

### Forced over-expression of the *PHOX2* genes results in increased *ALK* expression

To evaluate downstream effects of forced over-expression of each protein over the others, we transfected HeLa cells, which show the lowest transcription levels of the genes of interest, with *ALK*, *PHOX2A* and *PHOX2B* cDNAs expressing vectors.

**Figure 3:**
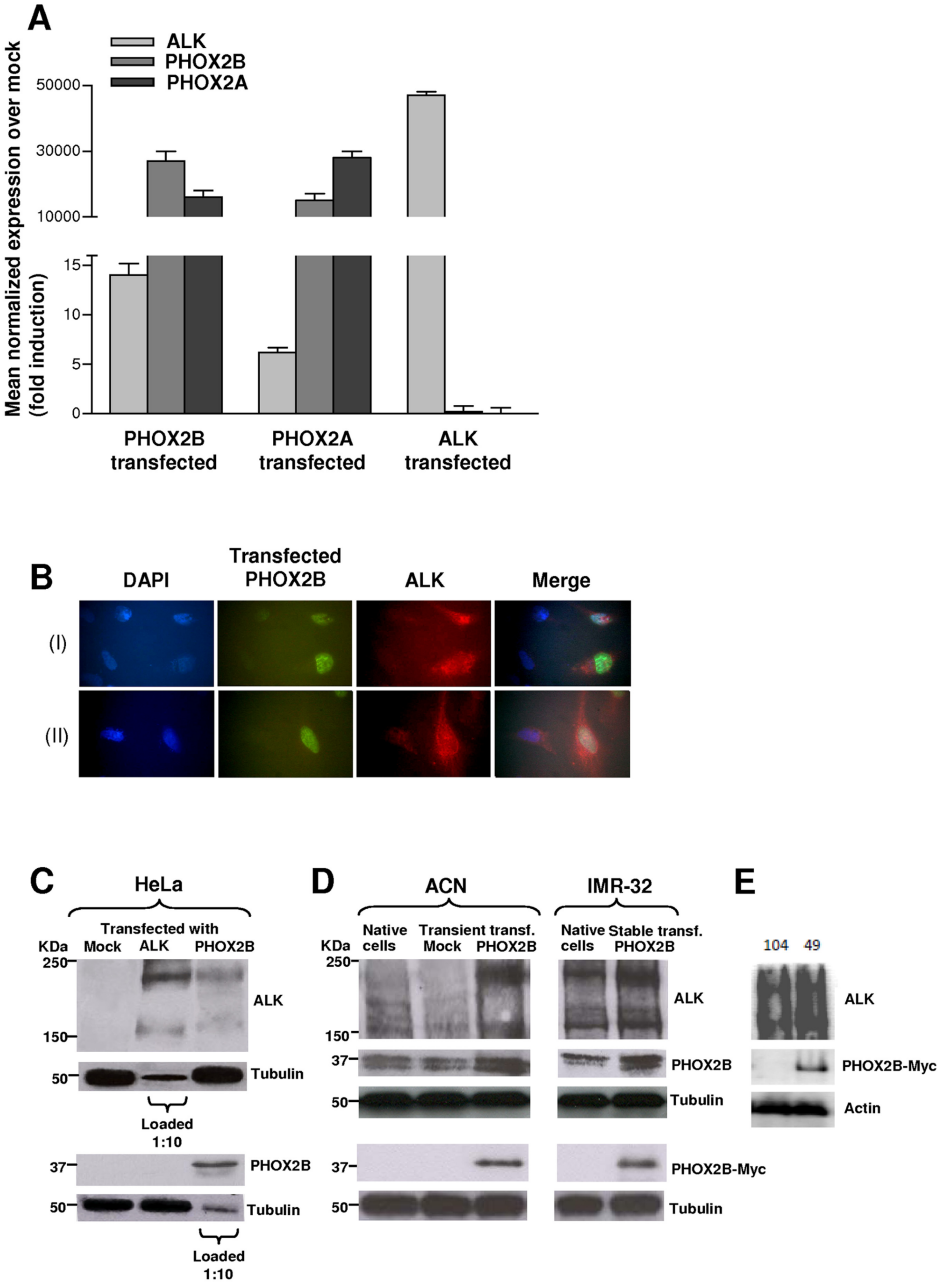
Forced over-expression of *ALK, PHOX2B* and *PHOX2A* in HeLa and NB cell lines. A) Transcription levels of the *ALK*, *PHOX2B* and *PHOX2A* genes were evaluated in HeLa cells 48 hours post-transfection with the corresponding gene-specific cDNA expressing vectors by real-time RT-qPCR. Besides the dramatic increase of gene transcripts by each respective transfectants, *ALK* expression results enhanced by the *PHOX2* genes over-expression. Values are the mean ± s.d. of N = 3 independent experiments performed in duplicate. B) Upper (I) and lower (II) lanes from immunofluorescence analysis report examples of HeLa cells transfected with the PHOX2B-Myc expression construct. From left to right images show DAPI stained cell nuclei (blue), staining for the PHOX2B-Myc protein (green), and staining for the ALK protein (red). The most distal image is the merge of the three nearby figures. C) Western blot evaluating protein amounts of ALK and PHOX2B in HeLa cells at 72 hours post-transfection with gene-specific cDNA constructs. A consistent transcript increment of each gene is observed but ALK starts to be expressed also following the forced expression of PHOX2B-Myc protein. Gel was loaded with 100 µg of total protein extracts except for evaluation of ALK in *ALK*-transfected cells and PHOX2B in *PHOX2B*-transfected cells, for which 1∶10 (10 µg) of protein extracts was loaded to avoid over-saturation of autoradiograph films. D) Western blot evaluating protein amounts of ALK in ACN cells (left) at 72 hours post-transfection with the PHOX2B-Myc construct, in a clone of IMR-32 cells stably expressing the same PHOX2B-Myc fusion protein (right). A marked increment in ALK expression is detected following PHOX2B-Myc expression in transient –transfected ACN cells compared to both native and mock-transfected cells and in a clone of IMR-32 cells, stably expressing PHOX2B-Myc, compared to native cells. An anti-cMyc antibody was specifically used to distinguish PHOX2B-Myc fusion protein from endogenous PHOX2B of NB cells (lower blots). E) Two stable IMR-32 clones, one negative (104) and one positive (49) for PHOX2B-Myc expression were analyzed for ALK expression (upper panels); expression of the fusion PHOX2B-Myc protein was assessed by Western Blot using anti –cMyc (middle panels) and the anti-actin antibodies (bottom panels).

Gene expression analysis carried out 48 hours post-transfection by Real-time RT-qPCR revealed that *PHOX2A* and *PHOX2B* constructs dramatically increased transcription levels of either genes (Figure 3A). Moreover, a relevant increment of *ALK* transcript was also obtained in *PHOX2B* and in *PHOX2A* transfectants (with about 14 and 6 fold induction over mock, respectively). On the other hand, over-expression of *ALK* had no effect on *PHOX2B* and *PHOX2A* transcript levels (Figure 3A), a result consistent with data obtained using siRNA-mediated knock-down and once again indicating an unidirectional expression regulation of *ALK* by *PHOX2* genes. Up-regulation of the ALK protein was also evaluated 48 hours after transfection with the *PHOX2B*-*Myc* plasmid, by immunofluorescence in permeabilized HeLa cells. As shown in Figure 3B, the intensity of ALK protein staining (red fluorescence) was much higher in cells expressing PHOX2B (green fluorescence) than in untransfected cells, which were positive only for the blue nuclear staining. These findings have been confirmed by Western blot analysis on total protein extracts at 72 hours post-transfection (Figure 3C) showing PHOX2B-mediated ALK up regulation not coupled to reciprocal ALK-mediated PHOX2B increase.

Finally, we transfected the NB cell line ACN, which shows very low expression levels of the three genes, with the *PHOX2B*-*Myc* plasmid. As already observed in HeLa cells, Western blot analysis revealed an increment of ALK protein at 72 hours post-transfection (Figure 3D, left). Similar results were also confirmed by evaluating ALK and PHOX2B protein amounts in a clone of IMR-32 stably expressing the same construct (Figure 3D, right), as well as by comparing two clones deriving from the same IMR-32 culture, expressing (# 49) and not expressing (# 104) PHOX2B/Myc (Figure 3E).

### *In silico* prediction of the *ALK* regulatory region and putative binding elements

In the light of the straightforward involvement of *PHOX2B* in NB development, and opposite to *PHOX2A* whose mutations never resulted in association with any neural crest derived tumor [14], we have focused on the former gene to deepen into molecular details of its regulative role on the *ALK* gene transcription.

**Figure 4:**
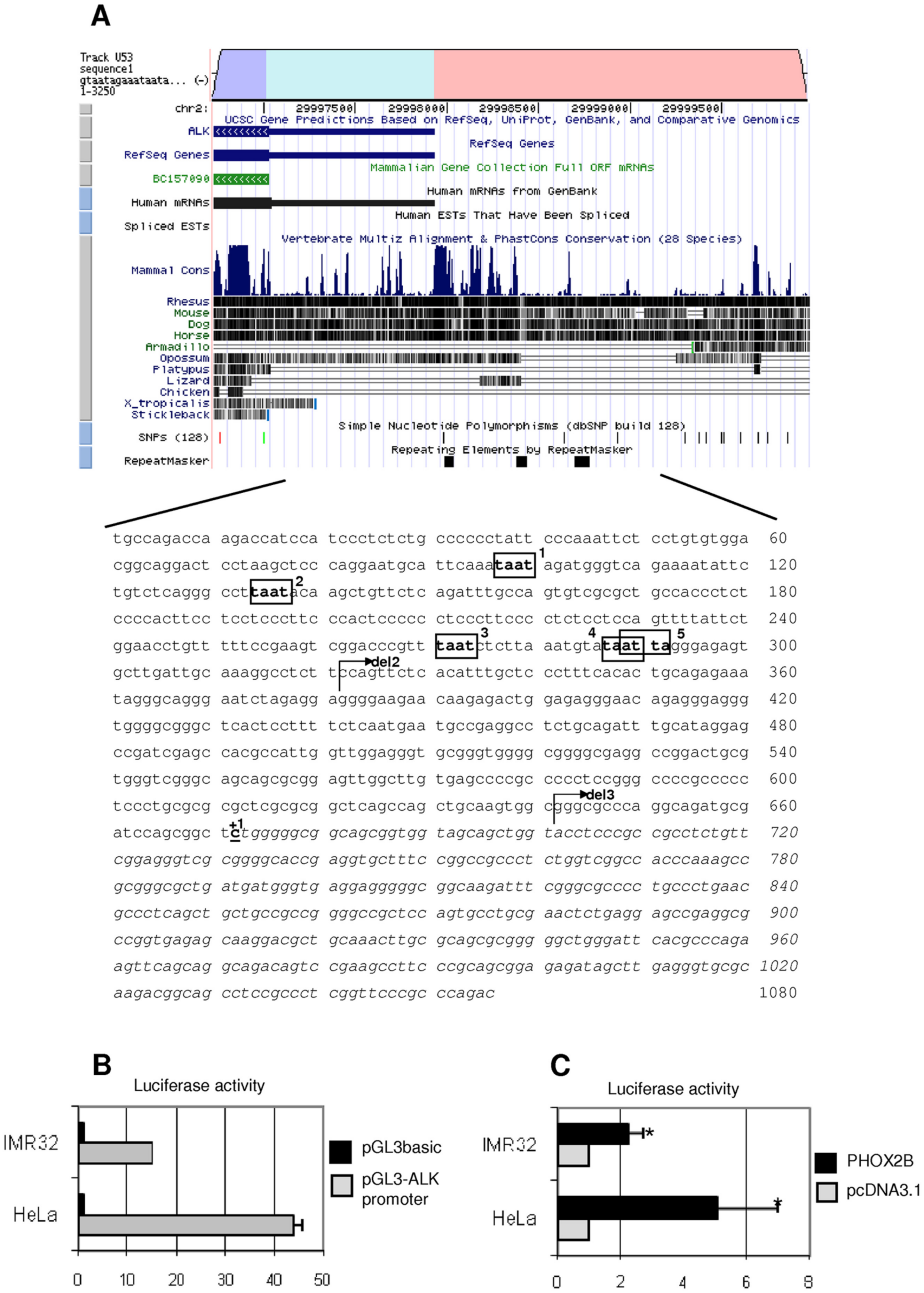
*In silico* representation of the subcloned *ALK* regulatory region. A) In the upper section of the figure, a portion of the *ALK* gene, as shown by the Vista Browser, is reported: the *ALK* coding region (dark grey), the predicted 5′ untranslated region (light grey), part of the 5′ regulatory region (black) and their levels of phylogenetic conservation (dark bars) are represented. In the lower part, the sequence corresponds to the part of the gene subcloned into the pGL3basic vector, containing 672 bp upstream the putative transcriptional start site (indicated with +1) and 384 bp of the 5′ untranslated region (shown in Italic). Moreover, the five ATTA sites are boxed and progressively named from 1 to 5. Arrows represent the start point of the deletion constructs. B) Activity of the pGL3basic-*ALK* promoter construct (grey bars) in HeLa and IMR-32 cell lines, expressed as fold induction with respect to the empty pGL3basic vector (black bars). Values are the mean ± s.d. of N = 3 independent experiments performed in duplicate. C) *ALK* promoter induction in HeLa and IMR-32 cells co-transfected with the PHOX2B expression plasmid and expressed as fold induction of the Luciferase activity with respect to cells transfected with the empty vector (pcDNA3.1, arbitrary value = 1) (*: *P*<0.05).

*In silico* analysis of about 3 kb sequence lying upstream of the *ALK* coding region was performed to search for conserved sequences, which were then identified in the portion encompassing from −1 kb to the *ALK* predicted transcriptional start site (GenomeVista, http://pipeline.lbl.gov/cgi-bin/GenomeVista). The Genomatix Matinspector software (http://www.genomatix.de), used to identify putative binding sites for transcription factors in this region, showed five sequences recognized by homeoproteins (ATTA boxes) we have named ATTA 1, 2, 3, 4 and 5 (Figure 4A). To investigate whether *PHOX2B* might take part in the transcriptional regulation of this region, a sequence spanning from about −672 bp up to +384 bp was amplified and inserted into the pGL3basic vector, upstream the *Luciferase* reporter gene. Basic activities of this promoter construct assessed with respect to the empty vector 24 hours after transfection in two different cell lines, the HeLa cells and IMR-32 cells, are reported in Figure 4B.

Once assessed a transcriptional activity driven by this portion of *ALK* promoter in both cell lines, the ability of PHOX2B to induce the *ALK* gene expression was then investigated. As shown in Figure 4C, co-transfection of an expression construct encoding for the PHOX2B-Myc fusion protein with a reporter plasmid containing the *Luciferase* gene under the transcriptional control of the *ALK* promoter showed, both in Hela and in IMR-32 cell lines, that PHOX2B is able to activate transcription of the *ALK* gene by regulating the portion under analysis of its promoter. Therefore, as in IMR-32 cells the PHOX2B-mediated trans-activation of the reporter gene was less relevant than in HeLa cells, likely due to the already high expression of endogenous PHOX2B (see Figure 3D), the further co-transfection experiments were carried out only in Hela cells.

### Interaction between PHOX2B and the *ALK* promoter

To deepen into PHOX2B-mediated trans-activation of the *ALK* promoter, direct binding between the ATTA 1, 2, 3 and 4/5 sequences and PHOX2B was investigated *in vitro* through electrophoretic mobility shift assays (EMSA), by incubating probes containing each of the ATTA sites with nuclear extracts from IMR-32 cells, expressing high levels of endogenous PHOX2B, or alternatively with the *in vitro* translated PHOX2B protein.

**Figure 5:**
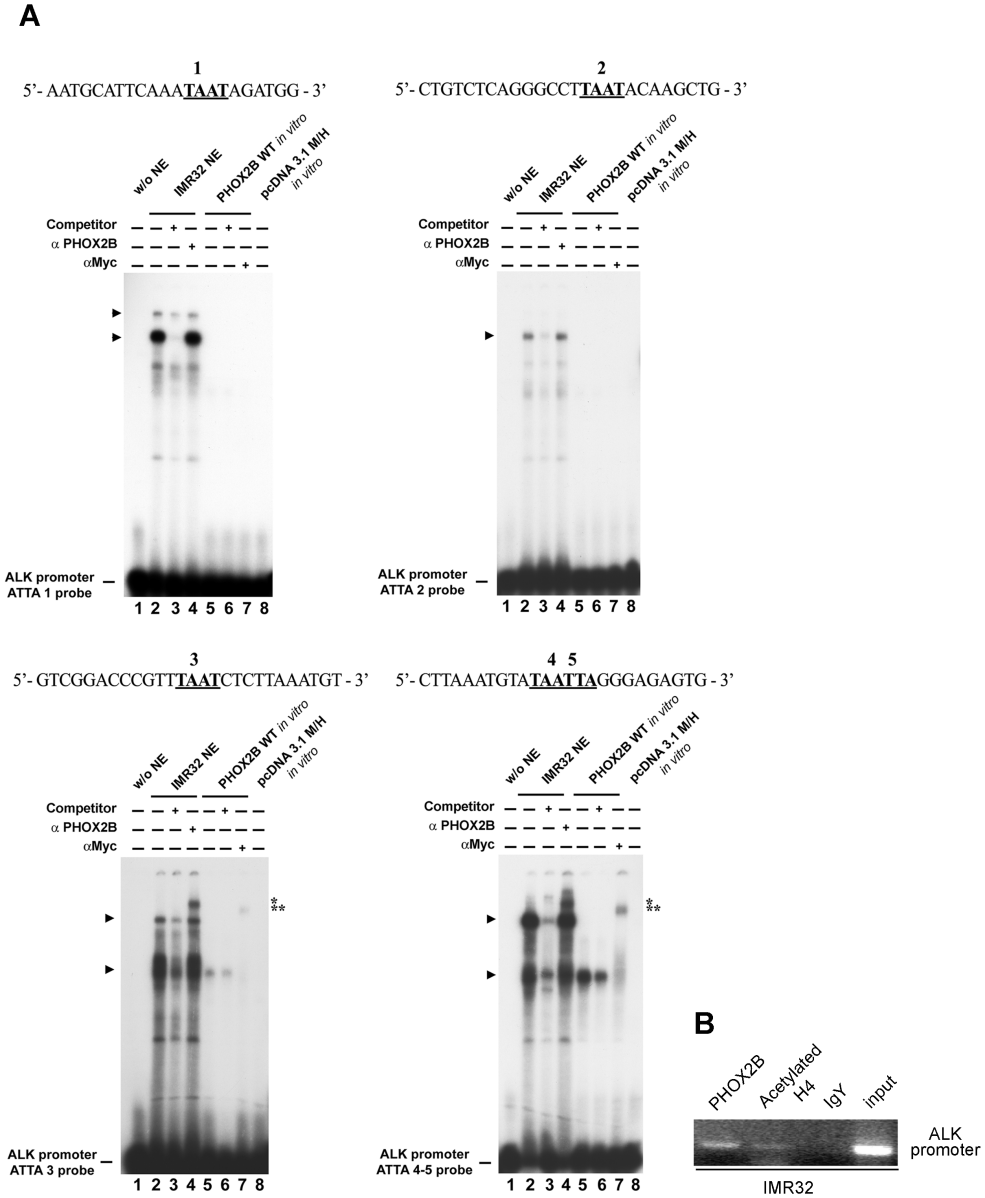
*In vitro* interaction of PHOX2B with the *ALK* promoter. A) EMSAs were performed using probes containing one of the ATTA sites of the region under analysis (ATTA 1, ATTA 2, ATTA 3 and the complex ATTA 4/5). Each labeled probe was incubated in the absence of nuclear extracts (lane 1), with IMR-32 nuclear extracts (lanes 2–4) or the *in vitro* expressed PHOX2B-Myc fusion protein (lanes 5–7). As negative control the oligonucleotides were also incubated with the *in vitro* reaction performed using the empty vector pcDNA3.1 M/H (lane 8). The competition experiments were performed in the presence of a molar excess of the unlabeled oligonucleotides (lanes 3 and 6). The anti-PHOX2B or the anti-c-Myc antibodies were added to the samples run in lanes 4 and 7, respectively. On the left, the arrows indicate the specific retarded bands detected; on the right, one or two asterisks indicate the supershifted complexes containing PHOX2B obtained by incubation of IMR-32 nuclear extracts with the anti-PHOX2B antibody (*) or the *in vitro* expressed protein with the anti-cMyc antibody (**), respectively. The free probes are shown at the bottom of the gels. B) ChIP assay. Chromatin extracted from IMR-32 cells was immunoprecipitated using the antibody against PHOX2B; pre-immune chicken IgY and the anti-acetylated histone H4 antibodies were used as negative and positive controls, respectively. The input represent 0,5% of the total chromatin extract. The precipitated DNA has undergone PCR amplification by using primers bordering the ATTA 3 and the ATTA 4/5 boxes in the *ALK* promoter.

As shown in Figure 5A, by incubating the IMR-32 extracts with probes including each of the ATTA 1, 2, 3 or 4/5 boxes we observed in any case formation of specific complexes (lanes 2), which disappeared in the presence of the corresponding unlabelled oligonucleotide (lanes 3). However, differently from what observed by probing the ATTA 1 and ATTA 2 sequences, the presence of PHOX2B inside the two complexes detected with ATTA 3 and ATTA 4/5 was confirmed by supershifted bands obtained following incubation of nuclear extracts with a PHOX2B specific antibody (lanes 4). Moreover, the fastest of two of the above specific complexes was obtained also by incubating the ATTA 3 and ATTA 4/5 probes with the *in vitro* translated PHOX2B-Myc fusion protein (lanes 5). While its specificity was assessed by the band disappearance in the presence of an excess of unlabelled oligonucleotide (lanes 6), presence of PHOX2B was confirmed by the supershifted band observed following incubation with a c-Myc antibody (lanes 7).

**Figure 6:**
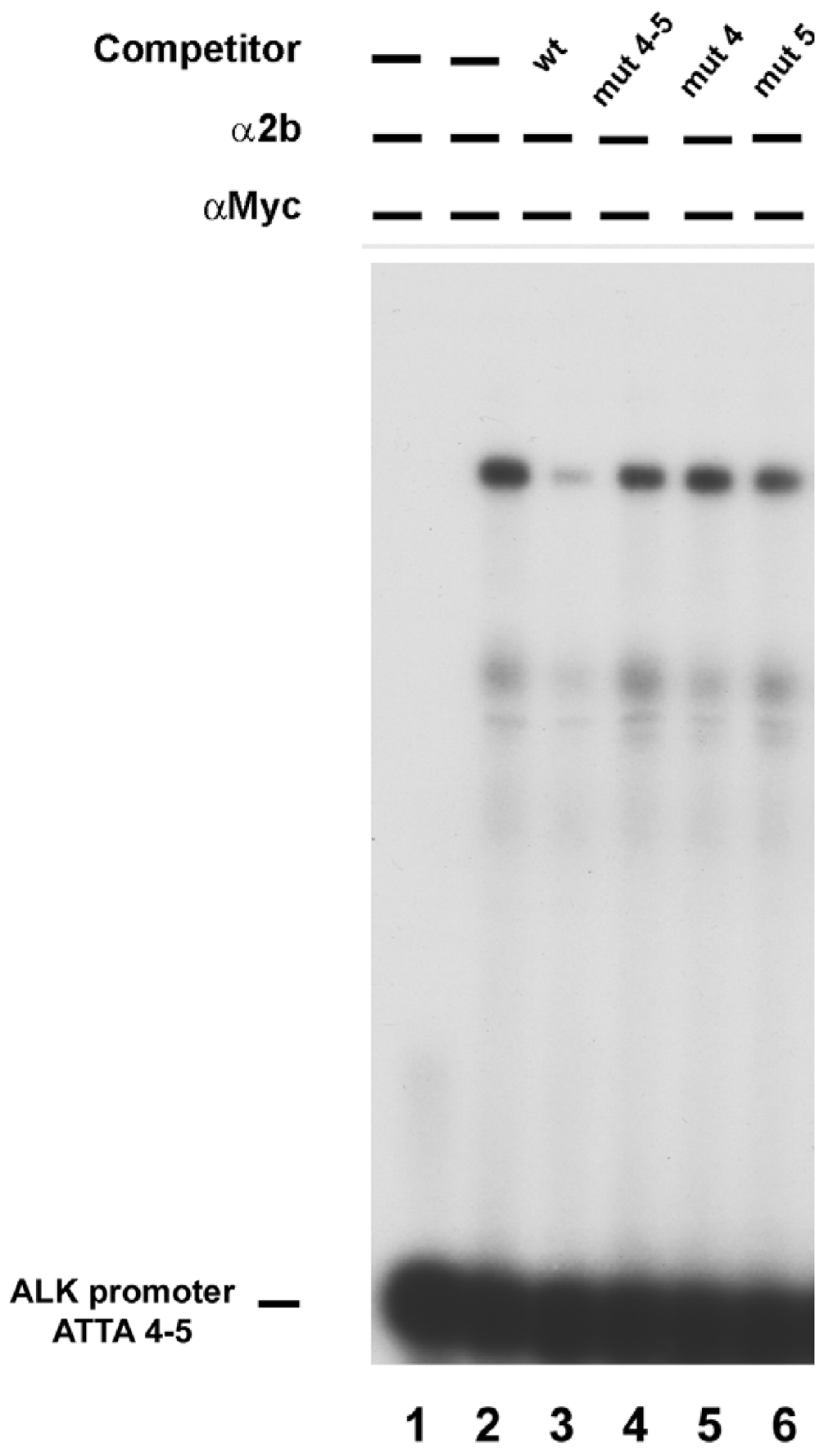
Effect of ATTA 4/5 disruption in competition of PHOX2B binding. IMR32 nuclear extracts were incubated with the ATTA4/5 probe (lane 2) and competition obtained by adding an excess of: a wild type (wt) probe (lane 3), a probe mutated in both ATTA 4 and ATTA 5 sites (lane 4) or in each of them (lanes 5–6). Incubation without nuclear extracts was regarded as negative control (lane 1).

Specificity of the ATTA4/5 sites was confirmed by performing EMSA following incubation of the probe carrying the ATTA4/5 boxes and competition assays with probes containing one or both the mutant ATTA sites; in particular, while the unlabelled ATTA 4/5 probe could efficiently compete for the PHOX2B binding, oligonucleotides carrying mutations in the ATTA box did not abolish DNA interaction with PHOX2B (Figure 6).

Finally, PHOX2B interaction with the *ALK* promoter was further confirmed by performing a ChIP assay. In particular, the chromatin extract from PHOX2B-expressing IMR-32 cells was incubated with the PHOX2B specific antibody, immunoprecipitated and the product obtained was amplified with primers surrounding the two ATTA 3 and ATTA 4/5 sites. Negative and positive controls of the reaction were obtained by incubating chromatin extracts with normal IgY and with hyperacetylated histone H4 specific antibody, respectively.

As shown in Figure 5B, presence of products amplified from immunoprecipitation obtained by using the anti-PHOX2B and anti-acetylated histone H4 antibodies suggests that in IMR-32 cells this is a transcriptionally active promoter region which, among others, can bind the PHOX2B transcription factor. Moreover, the absence of amplification of chromatin immunoprecipitated with pre-immune IgY confirmed the specificity of the assay.

### Effect of the ATTA 3 and ATTA 4/5 regions on PHOX2B-induced *ALK* trans-activation

**Figure 7:**
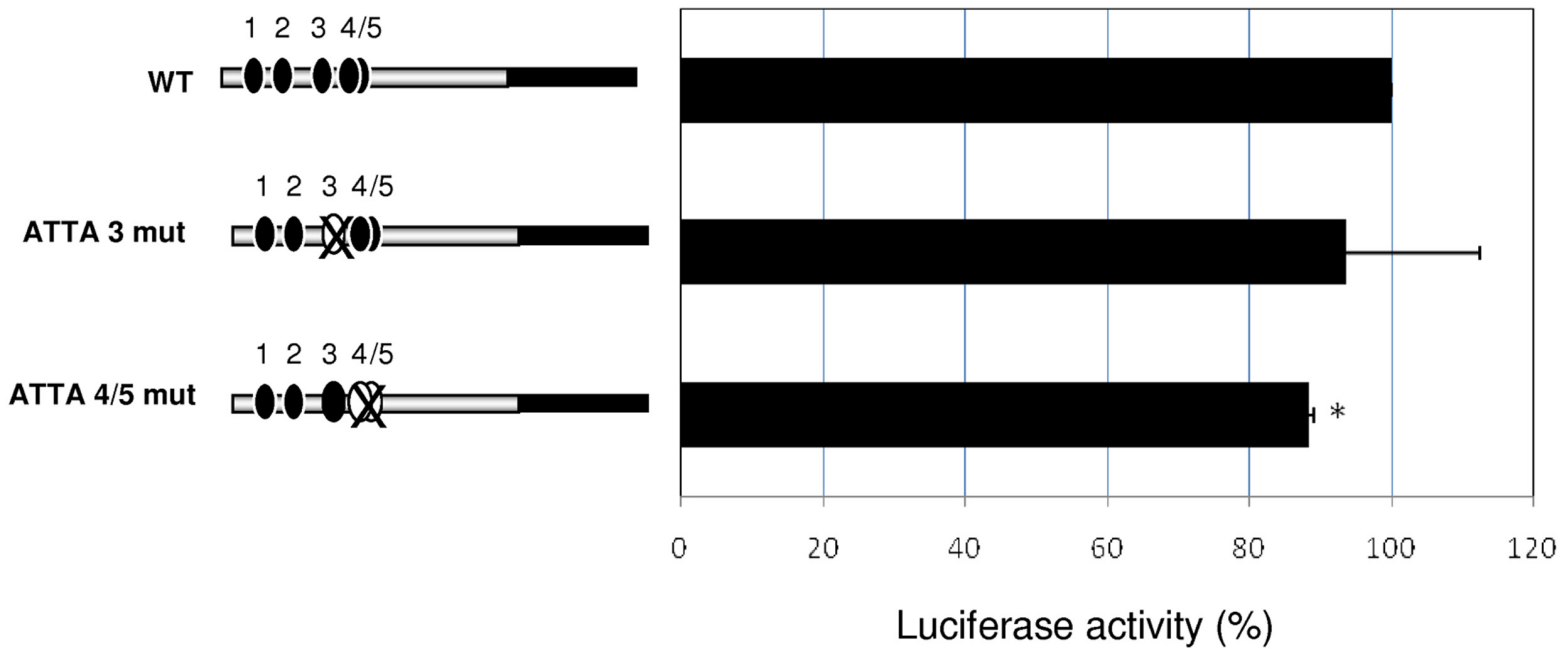
Effects of mutagenesis of ATTA 3 and ATTA 4/5 on the PHOX2B-mediated *ALK* trans-activation. Left side: schematic representation of the three constructs carrying all the ATTA boxes functional (wt, all four black circles), the ATTA 3 disrupted (ATTA 3 mut, one white circle) or both the ATTA 4 and 5 disrupted (ATTA 4/5 mut, two white circles). Right side: induction of the *ALK* promoter containing the mutant ATTA 3 and ATTA 4/5 in HeLa cells co-transfected with the PHOX2B expression plasmid are expressed as percentage of the Luciferase activity obtained by cells co-transfected with PHOX2B and the *ALK* promoter (wt) vectors (wt, arbitrary value = 100). Values are the mean ± s.d. of N = 3 independent experiments (*: *P*<0.05).

To verify whether only one or both the ATTA sites here identified to bind PHOX2B were also functionally active, thus mediating the PHOX2B trans-activation of this region of the *ALK* promoter, we co-transfected HeLa cells with each reporter construct of the *ALK* promoter, carrying mutant versions of ATTA 3, ATTA 4/5 or both elements, and the PHOX2B expression construct. As shown in Figure 7, mutagenesis of ATTA 3 could not significantly impair the ability of PHOX2B to activate *ALK* transcription, while disruption of the ATTA 4/5 induced a low but significant reduction of PHOX2B-mediated *ALK* trans-activation. As disruption of all the three ATTA boxes did not produce a more pronounced effect (not shown), we gathered that the only functional homeoprotein binding sequence responsible for PHOX2B-mediated trans-activation of the *ALK* promoter was the ATTA 4/5 box.

**Figure 8:**
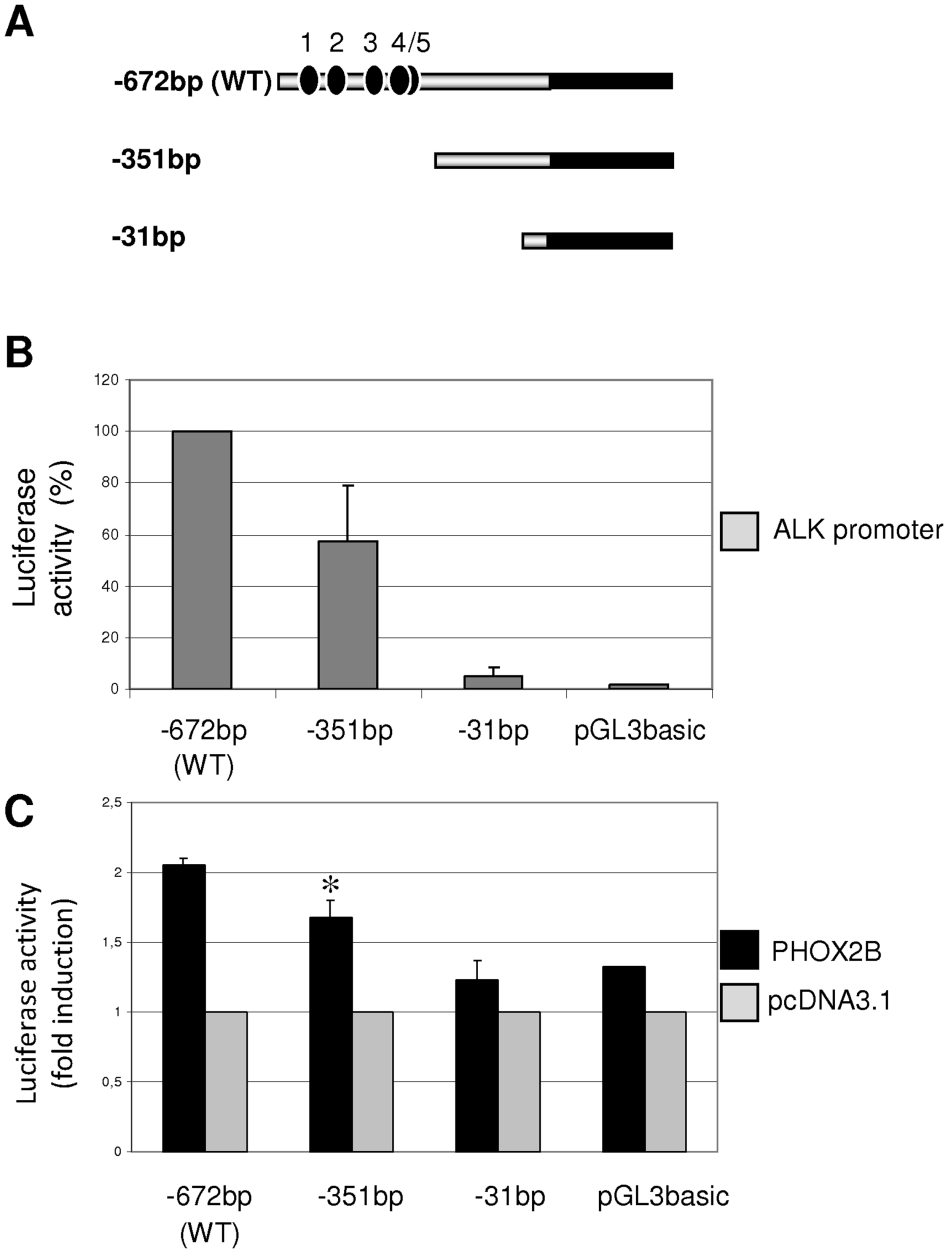
PHOX2B effect on the *ALK* promoter sequentially deleted plasmids. A) Schematic representation of deleted plasmid inserts, progressively shorter from the entire wt *ALK* promoter region considered (−671 bp), down to the so called deletion 2 (del2; −351 bp), and to the so called deletion 3 (del3; −31 bp) (see also figure 4A). The promoter (grey bar), the 5′UTR (black bar) and the ATTA boxes (black circles) are shown. B) Activity of the *ALK* promoter fragments, expressed as percentage of the activity of the wt construct. Values are the mean ± s.d. of N = 3 independent experiments performed in HeLa cells. C) PHOX2B-mediated induction of the *ALK* promoter deleted plasmids, expressed as fold increase of the Luciferase activity obtained with respect to the use of the empty vector (pcDNA3.1) on the wt promoter. Values are the mean ± s.d. of N = 3 independent experiments performed in duplicate in HeLa cells.

Finally, to investigate a possible indirect role of PHOX2B over different region of the *ALK* promoter, two deleted reporter constructs, lacking segments that include the ATTA sites, and characterized by shorter fragments of the *ALK* promoter (Figure 8A), were compared to the full length *ALK* promoter construct (wt) for their ability to mediate reporter expression in the presence of PHOX2B in HeLa cells. As shown in Figure 8B, removal of all the ATTA boxes markedly reduced the basal promoter activity, which was completely abolished only in the most proximal region. In addition, consistent with data obtained from mutagenesis of the ATTA boxes, co-transfection of *PHOX2B-Myc* expression plasmid and the *ALK*(−351 bp) promoter construct showed a lower trans-activation than that obtained using the entire *ALK*(−672 bp) promoter construct (Figure 8C). On the other hand, the activity of the shortest *ALK*(−31) fragment was not modulated by PHOX2B, suggesting that the residual effect of PHOX2B, likely indirect, is mediated by the −351 bp to −31 bp region of the *ALK* promoter.

## Discussion

Germline as well as somatically acquired mutations in the *PHOX2B* and *ALK* genes have been detected in both sporadic and familial NB cases [1]–[3], [10]. These mutations show an autosomal dominant inheritance with reduced penetrance in NB families and, while *ALK* mutations have clearly been demonstrated to act as “gain-of-function” mutations [3], [25]–[27], the molecular mechanism(s) underlying the effects of NB associated *PHOX2B* mutations is still to be determined. In fact, dominant negative effect, haplo-insufficiency or, at opposite, up-regulation of target genes may be considered equally possible consequences of *PHOX2B*-pathway alterations [2], [10], [12], [32]–[34]. Furthermore, wild-type as well as mutated transcripts of *ALK* and *PHOX2B*, and also the wild type transcript of its paralogue *PHOX2A*, have been found to be overexpressed in the vast majority of NB cell lines and tumor samples analyzed so far [3], [2], [14], [21], [27], [33], thus providing strong indications that up-regulation of these genes is involved in NB molecular pathogenesis. Accordingly, a very recent study has demonstrated that the disease is sustained by *MycN*-driven expansion of Phox2b expressing neuronal progenitors, in a transgenic mouse model of NB [35].

Activation of the *ALK* proto-oncogene is required for tumor transformation, through the induction of several downstream pathways which control key cellular processes such as cell-cycle progression, survival, cell migration and cell shaping [24]. As for other proto-oncogenes encoding receptor tyrosine kinases, ALK activation is physiologically achieved upon ligand and co-receptor binding but might be also triggered through different mechanisms such as DNA mutations, gene amplification or chromosomal translocations, as well as post-translational modifications. Remarkably, only a proportion of NB tumor samples with high level of *ALK* expression carries gene amplification and/or mutations [3], [21], [26]–[30], therefore, most of the *ALK* over-expression and its functional effects are uncharacterized and the underlying molecular mechanisms still undisclosed.

Herein, we report for the first time evidences of a direct role of *PHOX2B* in the transcriptional regulation of *ALK*, thus pointing not only at a combined role of these two genes in NB pathogenesis but also suggesting a possible synergistic and joined effect of *PHOX2B* and *ALK* in the development and/or maintenance of the sympathetic nervous system.

Furthermore, we have dissected a 1 kb region upstream of the *ALK* coding sequence, shown to be conserved and to contain several TAAT/ATTA boxes, two of which (ATTA 3 and ATTA 4/5) have been demonstrated to bind PHOX2B. Among the two homeoprotein recognition sequences, the ATTA 4/5 has shown to bind PHOX2B at a higher extent than ATTA 3, an observation in accordance with the decreased PHOX2B-mediated *ALK* trans-activation detected when this sequence is disrupted. Moreover, binding of the PHOX2B transcription factor to the *ALK* promoter regulatory region was assessed on chromatin immunoprecipitated from the PHOX2B-expressing IMR-32 cells by using a specific antibody. In the attempt to explain why mutations of the ATTA 3 and ATTA 4/5 sites did not completely abolish the PHOX2B ability to upregulate the *ALK* promoter activity, we have identified residual PHOX2B activity in the *ALK* promoter region spanning from −351 bp to −31 bp upstream the transcriptional start site. Though the binding to this region may represent an additional mechanism through which PHOX2B promotes *ALK* transcription, it may be also due to an indirect effect of *PHOX2B* over-expression on this proximal portion of the *ALK* promoter. Consistently, the most proximal 31 bp showed an unspecific activation, similar to that induced by the empty reporter vector.

Based on these observations, we can conclude that *ALK* is a novel PHOX2B target gene. Therefore, data herein reported add knowledge about the transcriptional cascade triggered by PHOX2B, its physiological role in the sympathetic nervous system and, relevantly for understanding the NB molecular pathogenesis, they prove that *ALK* and *PHOX2B* act in a same pathway which, once impaired or dysregulated, may affect the risk for NB development.

The increased *ALK* expression mediated by PHOX2B is in accordance with over-expression detected for both these genes in NB samples; however, while the non physiological *PHOX2B* up-regulation, alone or in concert with additional mechanisms, can easily account for increased *ALK* expression, the mechanisms leading to *PHOX2B* up-regulation are still unknown. Moreover, as a result of *PHOX2A* silencing and *PHOX2A* over-expression, a role of this protein in the *PHOX2B* transcription has to be assumed. Such a regulatory mechanism, already reported to be specific for noradrenergic differentiation in locus coeruleus [7], is novel for NB cells where it could take part to the cascade leading to the above described dramatic gene over-expression. Further investigations are also needed to clarify how the *PHOX2A* up-regulation in NB is achieved and whether it may have a role in NB pathogenesis, especially in the light of the lack of any *PHOX2A* mutation in NB samples and cell lines [14].

Taking into account the remarkable genetic heterogeneity of NB and its putative complex oligogenic inheritance [13], [22], the *PHOX2B*-mediated activation of *ALK* herein reported provides new insights in their common pathway(s), which may turn out useful for the identification of additional genes relevant to NB development. Finally, according to what proposed for ALK inhibition by targeted therapy [36], the new finding of a concurrent involvement of *ALK* and *PHOX2B* genes in NB initiation and progression opens new perspectives on the design of innovative therapeutic RNAi-mediated strategies to knock-down multiple target genes and is expected to have a fundamental impact for this ominous pediatric tumor, which is often refractory to conventional therapies.

## Materials and Methods

### Cell cultures and *in vitro* transfections

A panel of 13 NB cell lines, namely GI-LI-N and GI-ME-N [37], [38], HTLA-230 [39], ACN [40], LAN-1 [41], LAN-5 [42], IMR-5 and IMR-32 [43], SH-SY5Y [44], SK-N-SH, SK-N-MC and SK-N-BE [45], SK-N-AS [46], SK-N-BE(2c) [47] and the cervix carcinoma cell line HeLa [48] were cultured as previously described [49], [50].

All cell lines were tested for mycoplasma contamination, cell proliferation and morphology evaluation, both after towing and within four passages in culture.

Silencing experiments were optimized in 6-well plates (2×10^5^ cells) for SH-SY5Y, IMR-32 and HTLA-230 cell lines, and transfection performed on either adherent cells (forward transfection) or freshly harvested cells in suspension (reverse transfection), using Lipofectamine RNAiMAX (Invitrogen, San Diego, CA) and SilencerSelect® GAPDH siRNA-FAM labelled (fluorescein-labelled) (Ambion-Applied Biosystems, Austin, USA) at different molarity. Transfection efficiency was evaluated by either fluorescence microscopy or fluorescence activated cell sorter (FACS Calibur, Beckton Dickinson, San Jose, CA, USA). For each candidate gene 3 SilencerSelect® pre-designed gene-specific siRNAs were tested in parallel with a scrambled control (Silencer®Negative Control # 1, Ambion-Applied Biosystems), a housekeeping positive control (SilencerSelect® GAPDH siRNA, Ambion-Applied Biosystems), a blank with the transfection agent only and the native cells. The most effective silencers were thus selected for further experiments (siRNA ID #: s1271 for ALK, s1604 for PHOX2A, s17075 for PHOX2B, s6749 for TLX2, Ambion-Applied Biosystems). Transfection was set up at 100 nM siRNA and performed in either adherent cells (forward transfection), in serum free D-MEM without antibiotics and stopped after 14 hours with complete medium, or freshly harvested cells in suspension (reverse transfection), in complete D-MEM medium without antibiotics. The efficiency of gene silencing and downstream effect on other genes was evaluated at 24, 48 and 72 hours post-transfection by Real-time RT-qPCR and at 72 hours by Western blot.

HeLa cells (4,5×10^5^) were transiently transfected with 1,5 µg pcDNA3.1TOPO-*PHOX2A*, pcDNA3.1*Myc*-*PHOX2B* or pIRES-hrGFP-2a-*ALK* expression vectors in 60 mm diameter dishes with Fugene HD (Roche, Mannheim, Germany) using a 3∶1 Fugene/plasmid DNA ratio. Total RNA was isolated after 24, 48, 72 and 96 hours and analyzed for gene expression by Real Time RT-qPCR assays.

HeLa (1,5×10^6^ cells), ACN (2,5×10^6^ cells) and IMR-32 (3,2×10^6^ cells) cell lines were transfected with 7 µg pcDNA3.1TOPO-*PHOX2B* in 10 cm diameter dishes with Lipofectamine 2000 using a 5∶1 Lipofectamine/plasmid DNA ratio and cell lysates prepared 72 hours post-transfection for Western blot analysis.

All the gene silencing and gene over-expression experiments were performed in duplicate and repeated at least three times.

### Production of IMR-32-PHOX2B/Myc stable cell lines

IMR-32 cells were transfected with the above described PHOX2B expression construct encoding for the fusion protein PHOX2B/Myc. Two days after transfection, cell culture was added with 500 µg/ml G418 and maintained under selective conditions for three weeks to allow plasmid integration in the IMR-32 genome; then, survived clones were isolated and expanded in 48-wells plateswithculture medium added with 400 µg/ml G418, then expanded in 24-wells and subsequently in 6-wells plates. Western blot assays were performed by starting from an equal amount of cells for each condition.

### Total RNA isolation

To isolate total RNA from cell lines of interest we adopted a chemical extraction in combination with a silica-based membrane immobilization by using QIAzol and RNeasy mini and micro kit (QIAGEN, GmbH Hilden, Germany). A DNaseI treatment was also included for the removal of contaminating genomic DNA. RNA samples thus obtained were quantified by NanoDrop (Thermo Scientific, Rockford, USA). A quality control was assessed by 2100 Bioanalyzer (Agilent Technologies, Santa Clara, CA) using the RNA 6000 Nano chip (Agilent Technologies).

### Primer design and gene expression analysis by Real-time RT-qPCR

Based on the RefSeq annotations, gene specific primers were designed in the regions encompassing exon boundaries to generate a unique amplicon and tested for melting temperature and DNA folding (Table 1).

**Table 1:** Sequences of primers employed for real-time RT-qPCR.

Gene symbol	Forward primer	Reverse primer
ALK	5′-CTGTGGCTGTCAGTATTTGGAG-3′	5′-ACAGGTCAAGAGGCAGTTTCTG-3′
PHOX2B	5′-AGGGACCACCAGAGCAGTC-3′	5′-CTTGCGCTTCTCGTTGAGG-3′
PHOX2A	5′-TCGCTGAGACCCACTACCC-3′	5′-CCTGTTTGCGGAACTTGG-3′
TLX2	5′-CTAGCGGGACTCACCTTCC-3′	5′-CCCAGAGAAGGGCGAGA-3′
GAPDH	5′-GAAGGTGAAGGTCGGAGT-3′	5′-CATGGGTGGAATCATATTGGAA-3′
POLR2A	5′-GACAATGCAGAGAAGCTGG-3′	5′-GCAGGAAGACATCATCATCC-3′
POLR3F	5′-CCTACTTTCTCAGTGGTTTCATTG-3′	5′-AAAGGCATGTTCCACTCTCC-3′
NDUFB3	5′-GGGACACCATTAGAAACTATCCAG-3′	5′-CAAAGCCACCCATGTATCTCC-3′

Three human genes (*POLR2A*, *POLR3F*, *NDUFB3*), resulted to be homogeneously and uniformly expressed in our samples, were chosen as endogenous controls for data normalization, carried out by using BestKeeper software (http://www.gene-quantification.de/bestkeeper.html). Primers were designed using Primer 3 software (http://fokker.wi.mit.edu/primer3/input.htm) [51]. Once tested primer amplification efficiencies by a standard curve, triplicates of each cDNA sample (12,5 ng) were amplified in the iCycler (Bio-Rad Laboratories, Hercules, CA) following an initial denaturation at 95°C for 2 min, then 50 cycles at 95°C for 15 sec and 60°C for 30 sec. Melting curves were calculated between 55°C and 95°C with increment of 0.5°C every 15 sec. PCRs were repeated at least twice.

For relative quantification of gene expression in native cell lines, we prepared an equimolar pool of RNAs from eight normal tissues of different embryonic origin (adrenal gland, bladder, breast, brain, colon, lung, placenta and prostate) (FirstChoice® Human Normal Tissue Total RNA, Ambion) to be employed as reference sample.

In transfected cells, gene expression level was compared to appropriate negative controls (i.e. native cells, transfectant agent only, scrambled sample or empty vector).

Analysis of mRNA expression level of the target genes (*ALK*, *PHOX2A*, *PHOX2B*) and the positive control (*GAPDH*) has been carried out by a two-step real-time RT-qPCR using a random priming-based reverse-transcription (High Capacity cDNA Reverse Transcription Kit, Applied Biosystems, Foster City, CA) and SYBR® Green I binding dye (Platinum® SYBR® Green qPCR SuperMix-UDG, Invitrogen) according to the manufacturer's conditions. Data were analyzed by qGene software, implemented to correct for amplification efficiencies [52].

### Western blot analysis

Total cell lysates from native or transfected cells (SH-SY5Y: 1,5×10^6^, IMR-32: 3,2×10^6^, HTLA-230: 1,2×10^6^, ACN: 2,5×10^6^ and HeLa: 1,5×10^6^ cells) in 10 cm diameter dishes were prepared and analyzed by Western blot analysis as described earlier [53].

### Construction of human *ALK* promoter reporter plasmids

*pGL3basic-ALK promoter* - The region from −672 bp to +384 bp with respect to the *in silico* predicted *ALK* transcription start site (GenomeVista, http://pipeline.lbl.gov/cgi-bin/GenomeVista) was obtained by PCR amplification from genomic DNA by using the following couple of primers: K(F) 5′-TGCCAGACCAAGACCATCCA-3′ and K(R) 5′-GTCTGGGCGGGAACCGA-3′, subcloned in pCR2.1 vector (TOPO-TA cloning kit, Invitrogen), completely sequenced and subcloned by SacI-XhoI double digestion in the pGL3basic vector upstream of the *Firefly Luciferase* reporter gene (Promega, Madison, USA). Two deletion constructs were derived from this promoter construct by coupling K(R) with the following primers: K(del2) 5′-CCAGTTCTCACATTTGCTCCC-3′ and K(del3) 5′-GGGCGCCCAGGCAGATG–3′.

*ATTA boxes site-specific mutagenesis* - Starting from the pGL3basic-*ALK* promoter wild type, site-specific mutagenesis was performed by PCR to disrupt the ATTA 3 and ATTA 4/5 putative PHOX2B binding site by using the following primers: ALK-ATTA3mut 5′-GGACCCGTTTCGTCTCTTAAATG-3′ and ALK-ATTA3mut(R) 5′-CATTTAAGAGACGAAACGGGTCC-3′ for ATTA3, ALK-ATTA4/5mut 5′-CTCTTAAATGTATGCGTAGGGAGAG-3′ and ALK-ATTA4/5mut(R) 5′-CTCTCCCTACGCATACATTTAAGAG-3′ for ATTA4/5, respectively. In particular, each of the final PCR products of the mutagenesis was subcloned in the pCR2.1 vector (TOPO-TA cloning kit, Invitrogen), completely sequenced and subcloned by SacI-XhoI double digestion in the pGL3basic vector upstream of the *Firefly Luciferase* reporter gene (Promega, Madison, USA) to obtain the final plasmids.

### Luciferase assays

Transient transfections were performed in complete medium plating 1,5×10^5^ cells directly with the transfection mix and adding 750 ng PHOX2B expression construct with 250 ng pGL3basic-*ALK* promoter, or equimolar amounts of deleted reporter constructs, with 3 µl of FugeneHD (Roche).

The plasmid pRL-TK, expressing the *Renilla Luciferase* gene, was used as an internal control for each sample. Forty-eight hours after transfection, cells were assayed for Luciferase activity (Dual-Luciferase Reporter Assay System, Promega) using a TD-20/20 Luminometer following manufacturer's instructions.

### Immunofluorescence analysis

HeLa cells were transiently transfected with 1,5 µg pcDNA3.1Myc-PHOX2B expression vector and, after 48 hours, cells were washed, permeabilized and then incubated with an ALK-specific and a Myc-specific antibodies (Invitrogen) for 90 min and, after PBS washing, with a TRITC- and FITC conjugated secondary antibodies, respectively. Cells were then washed with PBS, fixed for 3 min with acetone:methyl alcool 1∶1, permeabilized for 15 min with 0,1% Triton-X-100/PBS and blocked for 5 min with 10%FBS/1%BSA/0,1%Tween20/PBS). After nuclei staining with DAPI (Roche), cells were analysed by fluorescence microscope (Zeiss Axiophot, Nikon ACT-U).

### Nuclear extracts and Electrophoretic Mobility Shift Assay (EMSA)

The IMR32 nuclear extracts and the *in vitro* expressed PHOX2B-Myc fusion protein were prepared as previously described [17]; the *in vitro* reaction was also performed with the empty vector (pcDNA3.1 M/H) to be used as negative control in the EMSAs. The EMSAs were performed accordingly to already published methods [54]. The oligonucleotides used in the EMSA experiments are indicated in Table 2.

**Table 2:** Oligonucleotides used in EMSA*.

ATTA1	5′ **gg**aatgcattcaaataatagatgg 3′
ATTA1 (C)	3′ acgtaagtttattatctaccca**g**t 5′
ATTA2	5′ ct**g**tctcagggccttaatacaagctg 3′
ATTA2 (C)	3′ gagtcccggaattatgttcgacaa**g**a**g** 5′
ATTA3	5′ **g**tc**gg**acccgtttaatctcttaaatgt 3′
ATTA3 (C)	3′ tgggcaaattagagaatttaca 5′
ATTA4/5	5′ **gg**cttaaatgtataattagggagagtg 3′
ATTA4/5 (C)	3′ atttacatattaatccctctcac**g**aac 5′
ATTA4 mut	5′ **gg**cttaaatgtacaattagggagagtg 3′
ATTA4 mut (C)	3′ atttacatgttaatccctctcac**g**aac 5′
ATTA5 mut	5′ **gg**cttaaatgtataattcgggagagtg 3′
ATTA5 mut (C)	3′ atttacatattaagccctctcac**g**aac 5′
ATTA4/5 mut	5′ **gg**cttaaatgtatgcgtagggagagtg 3′
ATTA4/5 mut (C)	3′ atttacatacgcatccctctcac**g**aac 5′

*Original or mutated ATTA sequences are underlined in each oligonucleotide.

Competition and supershift assays were carried out by pre-incubating the reactions with the appropriate amounts of unlabelled oligonucleotide and antibody anti-PHOX2B [17] or anti-c-myc antibody (Sigma).

### Chromatin immunoprecipitation assay (ChIP)

Chromatin from IMR-32 cells was prepared as manufacturer's instruction (ChIP-IT Express, Active Motif); a little portion of the supernatants was kept as “input” material (0,5% total chromatin). Cleared chromatin was incubated overnight at 4°C with anti-PHOX2B antibody [17] or anti-acetylated histone H4. As negative control chicken pre-immune chicken IgY (Santa Cruz Biotechnology) were used. Immunocomplexes were collected by magnetic beads. Chromatin was isolated by reversing crosslinking at 65°C for 2 hours, followed by proteinase K treatment and DNA purification (NucleoSpin Extract II; Macherey-Nagel). The genomic sequence of interest, including both ATTA 3 and ATTA 4/5 boxes, was amplified by PCR using primer K(del1) 5′- CCCCTCTCCTCCAGTTTTATTC -3′ with primer K(R)2 5′- TCCCTCTGTTCCCTCTCC -3′.

### Statistical Analyses

All *in vitro* data are from at least three independent experiments. Results are expressed as mean values ±95% Confidence Interval (CI) for quantitative variables and as numbers and percentages for qualitative ones. For continuous variables, the statistical significance of differential findings between experimental and control groups was determined by ANOVA with the Tukey's multiple comparison test. The correlation between categorical variables was assessed by the Pearson's coefficient test. ANOVA and Pearson's tests were performed by Graph-Pad Prism 3.0 software (Graph-Pad Software, Inc, El Camino Real, San Diego, CA). All tests were two-sided and a P-value<0.05 was considered as statistically significant.
